# Role of CHD chromatin remodelers in heart development

**DOI:** 10.1007/s12519-026-01049-y

**Published:** 2026-06-20

**Authors:** Si-Yu Sun, Zhi-Yu Feng, Wei Sheng, Guo-Ying Huang

**Affiliations:** 1 Research Unit of Early Intervention of Genetically Related Childhood Cardiovascular Diseases (2018RU002), Chinese Academy of Medical Sciences and Peking Union Medical College, Children’s Hospital of Fudan University, Shanghai 201102, China; 2 Pediatric Heart Center, Shanghai Key Laboratory of Birth Defects, Children’s Hospital of Fudan University, Shanghai 201002, China; 3 Department of Pediatrics, Peking Union Medical College Hospital, Chinese Academy of Medical Sciences and Peking Union Medical College, Beijing 100730, China; 4 Fujian Key Laboratory of Neonatal Diseases, Xiamen Children’s Hospital, Xiamen 361006, China

**Keywords:** Cardiac development, Chromatin remodeling, Chromodomain helicase DNA-binding protein, Congenital-heart disease

## Abstract

**Background:**

Chromodomain helicase DNA-binding (CHD) proteins are ATP-dependent chromatin remodelers that regulate chromatin accessibility, genome organization, and lineage-specific transcription during embryonic development. There are nine members of this family of proteins. Increasing genetic and functional evidence links mutations in multiple CHD genes to congenital-heart disease, arrhythmias, and cardiomyopathies. However, the stage-specific contributions of individual CHD-family members to cardiac development and functional maturation remain incompletely defined.

**Data sources:**

This narrative review summarizes studies retrieved from the PubMed database that investigate CHD chromatin remodelers in heart development and cardiac disease, with an emphasis on genetic models, developmental analyses, and recent multi-omics approaches.

**Results:**

Available evidence indicates that distinct CHD-family members contribute to cardiac development with different levels of support across human genetics, in vivo models, and stem-cell systems. CHD7 has the strongest combined evidence base, including human syndromic genetics and mouse lineage-specific studies, supporting roles in enhancer accessibility, second-heart-field or neural-crest programs, outflow-tract morphogenesis, and later cardiomyocyte maturation. CHD3 and CHD4, as ATPase subunits of the nucleosome remodeling and deacetylase complex, are supported mainly by mouse developmental studies and emerging human genetic data indicating functions in chamber specification, maintenance of lineage boundary, and developmental gene silencing. For CHD8, direct cardiac evidence remains limited. Available data suggest roles in cardiomyocyte survival, ventricular growth, sarcomeric organization, and metabolic homeostasis, but several mechanistic interpretations are inferred from stem-cell or non-cardiac systems. Direct cardiac roles of CHD1, CHD2, CHD5, CHD6, and CHD9 remain poorly defined.

**Conclusions:**

Current studies support a working model in which CHD remodelers shape enhancer activity, transcription-factor occupancy, and chromatin accessibility during cardiac morphogenesis and maturation. However, coordinated regulation across CHD-family members has not yet been directly established experimentally. Future progress will depend on integrative genetics, time-resolved multi-omics, and cardiac organoid/in vivo validation.

**Graphical abstract:**

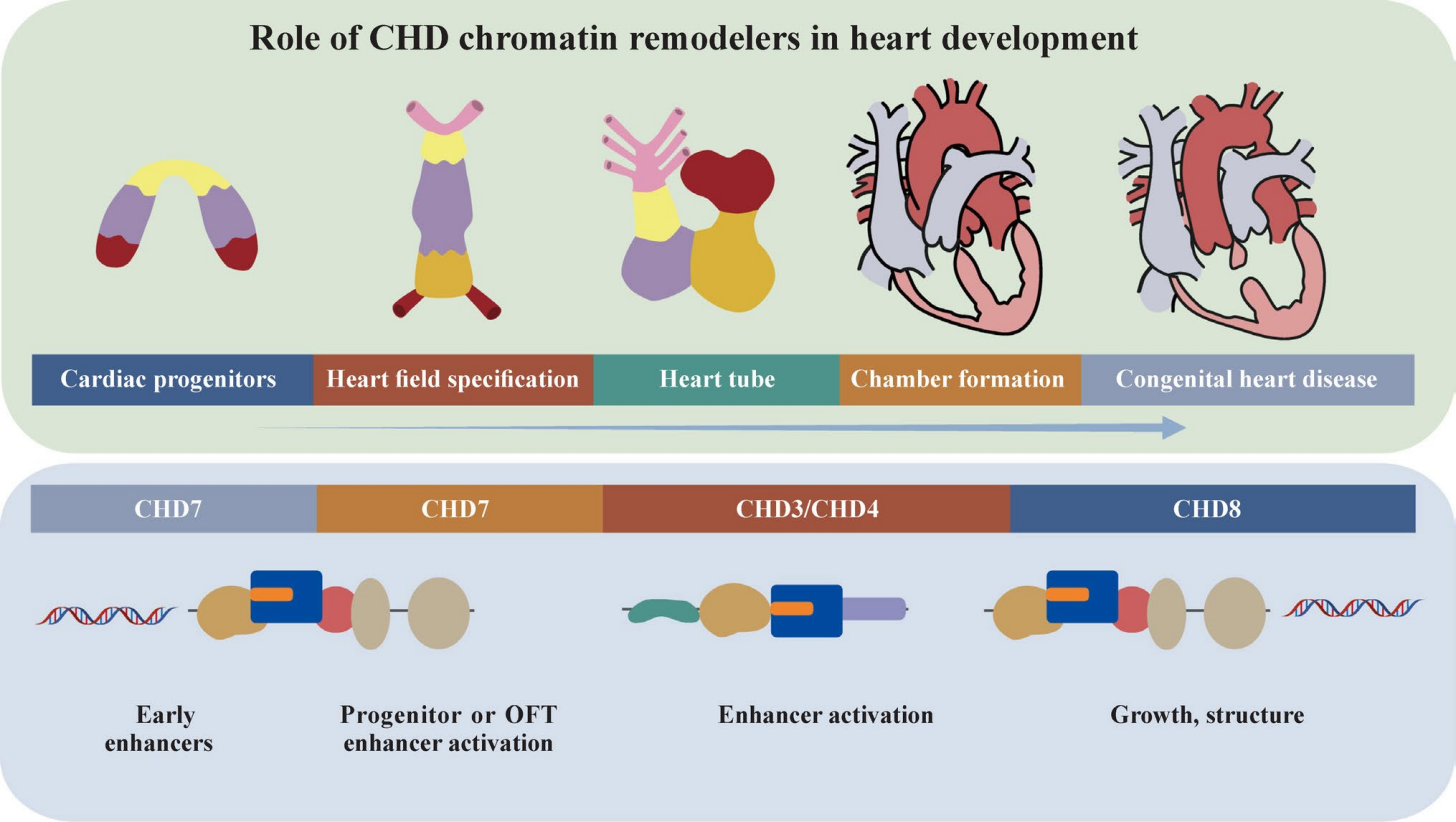

## Introduction

The mammalian heart is the first functional organ to form during embryogenesis, and its development proceeds through a series of highly coordinated events within narrow temporal windows [1–3]. Mesodermal cells migrate anteriorly during gastrulation to generate the cardiac crescent, which contains progenitors from the first-heart and second-heart fields. These populations contribute differentially to later cardiac structures, including the ventricles, atria, and outflow tract [4]. Subsequent looping, septation, and valve formation ultimately establish the mature four-chambered heart [5, 6].

These developmental transitions depend on precisely timed gene regulation. Classical cardiac transcription factors, including NK2 homeobox 5 (NKX2-5), GATA binding protein 4 (GATA4), T-box transcription factor 5 (TBX5), and myocyte enhancer factor 2 (MEF2)-family proteins, cooperate with signaling pathways such as bone morphogenetic protein (BMP), wingless/integrated (WNT), and NOTCH signaling pathways to specify lineage identity and coordinate morphogenesis [7–9]. However, the remarkable temporal and geographical specificity of cardiac gene regulation cannot be explained by transcription-factor binding alone. There is mounting evidence that chromatin architecture and enhancer accessibility represent an extra regulatory layer that controls the timing and location of transcriptional regulation [10–12].

Among ATP-dependent chromatin remodelers, the chromodomain helicase DNA-binding family has emerged as a central epigenetic regulator in heart development [12–15]. CHD proteins, which comprise nine family members, combine a sucrose non-fermenting 2 (SNF2)-like ATPase domain with chromodomains and additional accessory modules that enable context-specific interactions with histone marks, transcription factors, and chromatin-associated proteins. Through these activities, CHD remodelers modulate nucleosome positioning and transcriptional output in response to developmental cues.

In this review, we summarize current knowledge of the structural features and chromatin-remodeling mechanisms of the CHD family and discuss their roles in cardiac development and functional maturation. To enhance conceptual clarity, CHD remodelers are considered within the context of cardiac developmental biology, and evidence is distinguished according to its source. This includes human genetics, mouse conditional knockout models, stem-cell and organoid systems, and mechanistic inference from non-cardiac contexts. Within this framework, we highlight both the distinct and potentially cooperative functions of key CHD members and present the relationship between CHD7, CHD3/4-nucleosome remodeling and deacetylase (NuRD), and CHD8 as a working model for cardiac morphogenesis and maturation. Together, insights from human genetic studies and experimental model systems provide an emerging epigenetic perspective on congenital-heart disease.

## Heart development, cardiac morphogenesis, and congenital-heart disease

Cardiac development can be organized into a limited number of chromatin-sensitive transitions: heart-field specification, outflow-tract and septation morphogenesis, chamber differentiation, and postnatal cardiomyocyte maturation. This framing helps connect epigenetic regulation to concrete biological outcomes. Outflow-tract malalignment and great-artery defects reflect disturbed second-heart-field and neural-crest programs; atrioventricular septation and chamber-patterning defects reflect altered lineage fidelity within the myocardium and endocardial cushion system; and ventricular dysfunction or cardiomyopathy-like phenotypes reflect impaired structural, metabolic, or electrophysiological maturation.

**Fig. 1:**
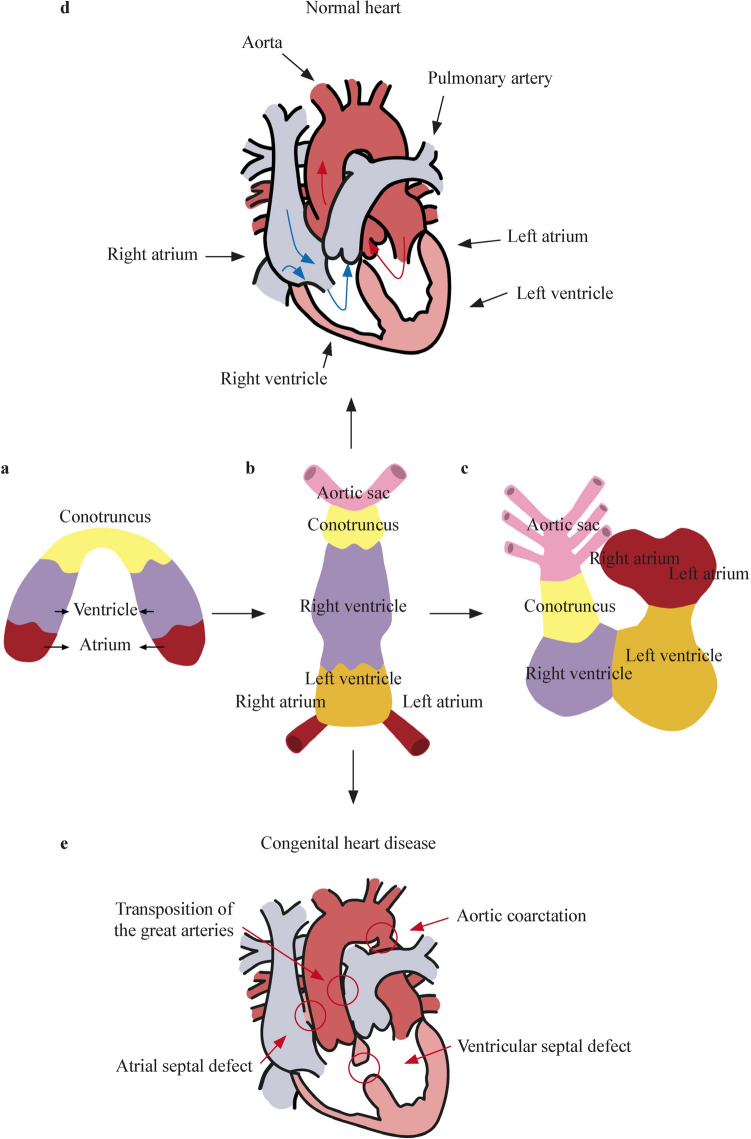
Normal heart development and congenital heart defects

During early embryogenesis, cardiac progenitors arise from anterior lateral plate mesoderm and form the cardiac crescent. The first heart field differentiates relatively early and contributes prominently to the left ventricle and portions of the atria (Fig. 1a), whereas the second-heart field remains proliferative and is progressively added to the arterial and venous poles, supporting outflow-tract elongation, right-ventricular development, and atrial expansion (Fig. 1b) [2, 5, 16, 17]. The linear heart tube then forms, loops rightward, and establishes the spatial architecture required for later chamber ballooning and regional specialization (Fig. 1c) [18, 19]. Septation and valve formation require coordinated integration of multiple lineages and tissue compartments. Endocardial cushion formation depends on epithelial-to-mesenchymal transition and extracellular-matrix remodeling, cardiac neural-crest cells contribute to outflow-tract septation and great-vessel patterning, and epicardial derivatives support fibroblast and coronary smooth-muscle development. By approximately the eighth gestational week in humans, the fundamental four-chamber cardiac architecture is established [2] (Fig. 1d). Structural cardiac abnormalities may arise from disruptions in proliferation, migration, differentiation, or signaling during these times. Failures in septation, outflow-tract alignment, or vascular remodeling lead to common congenital cardiac disorders, such as atrial and ventricular septal defects, transposition of the great arteries, and coarctation of the aorta [20]. Their pathogenesis includes altered extracellular-matrix interactions and intercellular communication in addition to transcription-factor malfunction (Fig. 1e).

Seen from this perspective, chromatin remodelers are not generic background regulators. They influence specific morphogenetic modules by shaping enhancer accessibility and transcription-factor occupancy in defined lineages [14, 21, 22]. This process-anchored view provides a more biologically coherent framework for interpreting CHD-associated phenotypes in congenital and postnatal heart disease.

## Structural features and chromatin-remodeling mechanisms of the CHD family

The chromodomain helicase DNA-binding family represents a conserved group of ATP-dependent chromatin remodelers defined by two hallmark domains: tandem chromodomains and an SNF2-like helicase ATPase domain [23]. The fundamental metabolic processes that underpin CHD function in a variety of biological situations are conferred by these structural modules. While the ATPase domain uses the energy of ATP hydrolysis to reposition, remove, or reconstruct nucleosomes, thereby dynamically regulating chromatin accessibility and transcriptional competence, the chromodomains allow CHD proteins to identify particular histone methylation states [15].

**Fig. 2:**
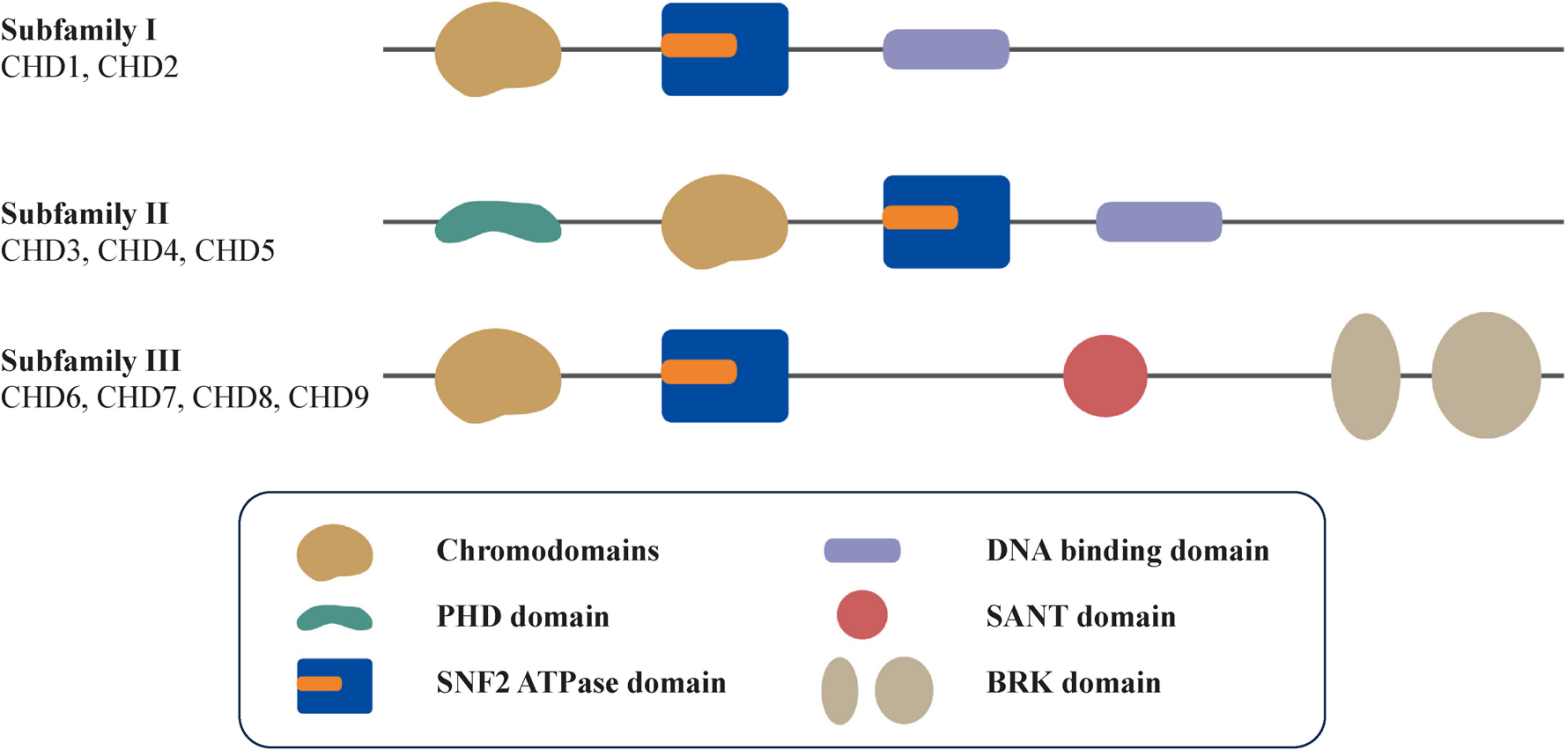
Schematic representation of all nine human CHD proteins divided by class, domain representation of all three CHD subfamilies as indicated in the text. *CHD* chromodomain helicase DNA-binding, *PHD* plant homeodomain, *SNF2* sucrose non-fermenting 2, *SANT* Swi3, Ada2, N-CoR, and TFIIIB

Individual CHD proteins contain extra regulatory domains that provide functional specificity, even though this fundamental design is conserved (Fig. 2). The CHD family is often split into three subfamilies based on domain makeup and biochemical function. Tandem chromodomains found in class I CHDs (CHD1 and CHD2) preferentially identify histone H3 lysine 4 di- and trimethylation, epigenetic markers linked to transcriptionally active promoters and enhancers [24]. CHD1 and CHD2 are typically linked to transcriptional activation, nucleosome turnover, and the preservation of open chromatin states, all of which are consistent with this binding preference. In multiple systems, these remodelers facilitate transcription initiation and elongation, supporting gene expression programs that require rapid and dynamic regulation [15, 25, 26]. Plant homeodomain fingers, which identify weakly methylated or context-specific histone modifications, set apart class II CHDs (CHD3, CHD4, and CHD5) [27, 28]. Members of this subfamily are most prominently associated with transcriptional repression through their incorporation into the NuRD complex [29]. As the main ATP-dependent motor in NuRD, CHD4 coordinates nucleosome repositioning via histone deacetylase 1/2-mediated histone deacetylation [27, 30]. During development and differentiation, NuRD creates compact chromatin states and enforces context-specific gene silencing in conjunction with other subunits, such as methyl-CpG binding domain protein 2/3, GATA zinc finger domain containing 2A/2B, retinoblastoma binding protein 4/7, and metastasis associated protein. The largest and most architecturally complex members of the family are class III CHDs (CHD6, CHD7, CHD8, and CHD9) [31, 32]. These proteins enable extended interactions with transcription factors, co-activators, and chromatin-associated proteins by integrating various regulatory areas outside of the core chromodomains and ATPase module. Among them, CHD7 and CHD8 have been most intensively studied, particularly in the context of human developmental disorders. Rather than functioning solely as repressors or activators, Class III CHDs often act at distal regulatory elements, modulating enhancer accessibility and transcriptional output in a highly context-dependent manner.

Despite this structural and functional diversity, a unifying feature of all CHD proteins is their ability to modulate nucleosome positioning and density. Nucleosomes represent the fundamental units of chromatin organization, and their spatial arrangement directly determines DNA accessibility. By sliding nucleosomes along DNA or transiently removing and reassembling them, CHD remodelers enable precise temporal and spatial control of gene expression [23, 33, 34]. Importantly, CHD proteins rarely act in isolation; rather, they function within a broader chromatin-remodeling network that includes switch/sucrose non-fermenting (SWI/SNF; also known as BRG1/BRM-associated factor, BAF), imitation switch (ISWI), inositol-requiring 80 (INO80), polycomb repressive complexes (PRC), and cohesin complexes [35]. Through cooperation and competition with these systems, CHD remodelers contribute to higher-order chromatin architecture, enhancer–promoter communication, and lineage-specific transcriptional landscapes.

## Roles of CHD chromatin remodelers in heart development and cardiac disease

For cardiac developmental biology, currently available data can be organized around three morphogenetic modules [36, 37]. First, outflow-tract morphogenesis and great-artery alignment are most strongly linked to CHD7 through cardiac neural-crest and second-heart-field enhancer regulation. Second, atrioventricular septation and chamber differentiation are most strongly linked to CHD4/NuRD through maintenance of lineage fidelity and transcription-factor-dependent chromatin tuning in myocardial development. Third, ventricular growth and postnatal functional maturation represent the domain in which CHD8 is emerging as a candidate regulator, although the direct evidence base remains more limited (Table 1).

**Table 1:** Chromatin-remodeling factors directly related to heart development

CHD member	Cardiac lineage	Morphogenetic module	Representative lesion	Chromatin mechanism	Functional phenotype	Reference
CHD7	Cardiac progenitors; second-heart field; cardiac neural crest; cardiomyocyte maturation	Outflow-tract morphogenesis; great-artery alignment; enhancer control during progenitor deployment	Conotruncal defects, arch anomalies, septation defects; later metabolic/electrophysiologic abnormalities in hypomorphic models	Enhancer co-occupancy with ISL1 and related factors; chromatin accessibility control at cardiogenic regulatory elements	Human syndromic genetics; mouse lineage-specific in vivo data	[12, 21, 22, 38, 39]
CHD4 (NuRD)	Cardiac progenitors; chamber myocardium; atrial cardiomyocytes	Chamber specification; atrioventricular septation; lineage-boundary maintenance	Embryonic lethality, septation defects, ventricular malformations, atrial/conduction phenotypes	NuRD-mediated repression of non-cardiac programs; context-dependent TF-guided remodeling	Mouse developmental genetics, emerging human genetics	[13, 14, 42]
CHD3 (NuRD-related)	Overlapping with CHD4	Possible compensatory/parallel control of lineage restriction	Cardiovascular involvement reported in syndromic settings; direct cardiac phenotype unresolved	Shared NuRD ATPase architecture; inference from related systems	Indirect human genetics, inference from non-cardiac systems	[31, 41, 43, 44]
CHD8	Early myocardium; ventricular myocardium; postnatal maturation	Ventricular growth; structural and metabolic maturation	Reduced proliferation/survival, altered sarcomeric organization, mitochondrial dysfunction; possible cardiomyopathy susceptibility	Possible p53-linked growth control and regulation of structural/metabolic gene programs	Impaired ventricular maturation; defective mitochondrial metabolism	[45–49]

*CHD* chromodomain helicase DNA-binding, *NuRD* nucleosome remodeling and deacetylase, *TF* transcription factor

### CHD7: enhancer-centric control of cardiac morphogenesis and functional maturation

Among CHD-family members, CHD7 currently has the strongest integrated cardiac evidence base, combining human syndromic genetics with mouse lineage-specific developmental studies [12, 38, 39]. Pathogenic variants in CHD7 are the principal genetic cause of CHARGE syndrome, a multisystem developmental disorder frequently accompanied by complex congenital-heart defects, particularly involving the outflow tract [12]. Consistent with the human genetics, neural-crest-specific deletion of *Chd7* in mice causes severe outflow-tract malformations and perinatal lethality, providing direct in vivo evidence for a requirement in cardiac neural-crest-derived morphogenesis [38, 39].

Mechanistically, CHD7 functions as an enhancer-associated remodeler that cooperates with lineage-defining factors such as ISL LIM homeobox 1 (ISL1) to modulate chromatin accessibility at cardiogenic regulatory elements [12]. Direct evidence from in vivo mouse models and in vitro developmental systems (stem-cell-based differentiation models) supports its roles in second-heart-field formation, neural-crest-associated patterning, and enhancer-dependent regulation of cardiac developmental genes. However, the strength of evidence differs across phenotypes: support for outflow-tract morphogenesis is strong, whereas support for later metabolic or electrophysiological functions derives in part from hypomorphic alleles and model-specific analyses.

Gene dosage sensitivity is a recurring theme in CHD7 biology. Loss-of-function alleles typically produce major developmental defects, whereas hypomorphic missense variants can reveal later-onset phenotypes, including cardiomyopathy-like remodeling, altered mitochondrial gene expression, and electrophysiological abnormalities [39]. These observations suggest that CHD7 links early morphogenesis with later functional competence, but they do not imply that every proposed downstream pathway is equally established across species and systems.

### CHD4 and CHD3: NuRD-mediated lineage restriction and context-dependent activation

CHD4 and CHD3 serve as the ATP-dependent remodeling cores of the NuRD complex [31, 40, 41]. While CHD4 has been more extensively studied in cardiac contexts, CHD3 is increasingly recognized as a functionally relevant and partially overlapping NuRD ATPase with distinct regulatory features [41]. During cardiogenesis, CHD4–NuRD enforces lineage fidelity by repressing inappropriate transcriptional regulation, thereby stabilizing cardiac progenitor identity and supporting orderly chamber differentiation. Genetic disruption of *Chd4* results in embryonic lethality and is associated with atrioventricular septation defects and ventricular malformations. NuRD-mediated repression is particularly critical during transitions between progenitor states and differentiated cardiomyocytes, where premature or ectopic gene activation would destabilize developmental trajectories. Importantly, NuRD activity in the heart is not uniformly repressive. In atrial cardiomyocytes, CHD4 can be recruited by transcription factors such as T-box transcription factor 5 (TBX5) to specific genomic loci, where it paradoxically increases local chromatin accessibility and promotes atrial gene expression [42]. This context-dependent switch between repression and activation highlights the plasticity of CHD4-mediated remodeling in fine-tuning atrial identity and electrophysiological homeostasis.

CHD3, although less well-characterized in cardiac development, shares high structural similarity with CHD4 and participates in NuRD complexes with overlapping but non-identical subunit compositions [15, 41]. Emerging evidence from developmental and disease models suggests that CHD3 contributes to transcriptional restraint and chromatin compaction in differentiating tissues [43, 44]. Given the tight requirement for temporal repression during cardiomyocyte maturation—particularly for non-cardiac or proliferative gene regulation—CHD3 is likely to act as a complementary NuRD ATPase in the heart. Pathogenic variants in CHD3 cause neurodevelopmental syndromes with frequent cardiovascular involvement, suggesting that CHD3-dependent NuRD dysfunction may contribute to congenital-heart disease through mechanisms analogous to, but distinct from, CHD4 loss [31].

CHD4 and CHD3 are related ATPase subunits of the NuRD complex, but their cardiac evidence bases are not equivalent. CHD4 is supported by direct mouse developmental studies and emerging human genetic evidence, whereas CHD3 has more limited direct cardiac support and remains partly inferential in the heart [13, 14, 31, 41, 42]. CHD4–NuRD contributes to repression of non-cardiac transcriptional regulation during cardiogenesis and thereby helps preserve lineage fidelity within the developing myocardium.

Importantly, NuRD activity is context-dependent rather than uniformly repressive. A concrete cardiac example is CHD4 recruitment by transcription factors, such as GATA4, NKX2-5, or TBX5, through which chromatin remodeling can support appropriate cardiac gene expression while still restraining ectopic lineage regulation [13, 42]. This point addresses the broader principle that CHD proteins rarely act in isolation: their functional specificity emerges through combinatorial interactions with transcription factors, deacetylases, and regulatory DNA elements.

Direct developmental studies implicate CHD4 in chamber differentiation, atrioventricular septation, ventricular patterning, and electrophysiological homeostasis. Human pathogenic CHD4 variants have also been associated with ventricular noncompaction and multisystem developmental phenotypes that include congenital cardiac abnormalities [14]. These data support a substantial role for CHD4 in cardiac morphogenesis, although specific lesion penetrance likely depends on variant class and developmental context.

With respect to CHD3, partial redundancy or compensation with CHD4 is plausible but remains unproven in cardiac development. CHD3 shares structural similarity with CHD4 and participates in NuRD assemblies with overlapping but non-identical subunit compositions [31, 41]. Cardiovascular findings have been reported in CHD3-associated neurodevelopmental syndromes, but direct mechanistic evidence in the heart remains limited. Simultaneous perturbation of CHD3 and CHD4 will be required to determine whether CHD3 and CHD4 act redundantly, sequentially, or in lineage-restricted parallel pathways during early heart development.

### CHD8: coordinating proliferation, structural assembly, and metabolic maturation

CHD8 remains an emerging cardiac regulator. Direct cardiac evidence currently suggests roles in cardiomyocyte proliferation or survival during early development and in aspects of ventricular structural and metabolic maturation, but the evidence is less extensive than that supporting CHD7 or CHD4 [13, 21, 22, 40]. Available data indicate that CHD8 can influence p53-associated growth control, sarcomeric organization, and mitochondrial homeostasis in cardiac or cardiomyocyte-like systems. However, many mechanistic interpretations still rely on stem-cell, neurodevelopmental, or other non-cardiac studies [45].

Current evidence regarding the role of CHD8 in the heart can be interpreted at three distinct levels [45–49]. First, direct observations from cardiac studies have reported ventricular growth abnormalities, altered sarcomeric organization, and impaired mitochondrial or contractile phenotypes in experimental model systems in mice. Second, mechanistic insights from other biological contexts suggest broader functions for CHD8 in regulating chromatin accessibility, transcriptional buffering, and haploinsufficient growth-related pathways. Third, these findings collectively raise the possibility that CHD8 dysfunction may contribute to susceptibility to cardiomyopathy or postnatal ventricular dysfunction. However, this hypothesis remains to be rigorously tested in dedicated cardiac models.

The postnatal maturation context is particularly relevant, because mammalian cardiomyocytes undergo a transition from predominantly glycolytic fetal metabolism toward mitochondrial oxidative phosphorylation and fatty-acid-supported energy production after birth. Structural maturation of sarcomeres, increased mitochondrial content, and metabolic rewiring are tightly coupled processes. Available data suggest that CHD8 may participate in this transition by helping maintain transcriptional regulation required for contractile organization and mitochondrial competence [45].

Collectively, CHD7, CHD3/CHD4, and CHD8 define distinct but interlocking chromatin-remodeling axes that orchestrate heart development and functional maturation. CHD7 governs enhancer activation and developmental competence, CHD3/CHD4 enforce lineage restriction while permitting context-specific activation, and CHD8 integrates proliferation, structure, and metabolism. Disruption of any component destabilizes the epigenetic landscape of the heart, providing a unifying framework for understanding congenital-heart disease and cardiomyopathy as disorders of chromatin regulation.

### Other CHD-family members and future directions

For CHD1, CHD2, CHD5, CHD6, and CHD9, direct cardiac evidence remains limited. Based on known domain functions and roles in other tissues, CHD1 and CHD2 likely facilitate early lineage commitment and transcriptional activation [23–26, 50, 51]; CHD5, recognized for stabilizing compact chromatin, may help maintain boundaries of sarcomeric gene expression [28, 34]; CHD6, linked to DNA-damage response and metabolic stress, may influence cardiomyocyte stress tolerance and repair [52–54]; and CHD9 remains poorly defined with sparse reports of tissue-specific functions. These hypotheses require systematic validation through cardiomyocyte-specific in vivo models, stem-cell differentiation systems, and time-resolved single-cell/spatial omics.

### Human genetics and clinical phenotype context

Human genetic evidence is strongest for CHD7. Heterozygous pathogenic variants cause CHARGE syndrome, and cardiac phenotypes are usually syndromic rather than isolated. Reported lesion spectra include outflow-tract defects, aortic-arch anomalies, septal defects, and more complex congenital presentations [21, 22]. In this setting, variable expression and incomplete penetrance are clinically important, and both variant class and broader developmental context likely influence phenotype severity.

CHD4 also has direct human cardiac relevance. Pathogenic variants have been associated with multisystem developmental disorders and, in selected cases, ventricular noncompaction or other congenital cardiac abnormalities [14]. The current literature suggests a spectrum in which missense or hypomorphic alleles may produce distinct outcomes from clear loss-of-function variants, but the dataset remains too limited to define firm genotype–phenotype rules.

CHD3 has more limited direct cardiac evidence in humans. Cardiovascular findings are reported in some CHD3-related neurodevelopmental syndromes, yet penetrance of cardiac phenotypes appears lower and mechanistic linkage to primary cardiac developmental processes is less well-defined [44]. CHD8, by contrast, is a well-established haploinsufficient neurodevelopmental gene, but direct evidence that specific CHD8 variants consistently cause congenital or isolated postnatal cardiac disease remains sparse [46, 47, 55]. At present, CHD8 should be regarded as a genetically plausible candidate gene for cardiac phenotypes, but its clinical relevance in the heart remains incompletely defined.

Overall, the human genetics literature supports a graded model in which CHD7 has the most robust association with syndromic congenital-heart disease, CHD4 has growing direct relevance to cardiac phenotypes, and CHD3/CHD8 remain either less penetrant or less clearly defined from a cardiovascular perspective.

## Evidence from studies on the stage-specific functions of CHD chromatin remodelers in cardiac development

Lineage specification, morphogenesis, and increasing cardiomyocyte differentiation and maturation are only a few of the closely controlled phases of cardiac development that call for exact epigenetic regulation. There is growing evidence that these processes involve CHD chromatin remodelers [12–14, 21, 22, 39, 45, 48]. It is important to remember that, rather than systematic studies of coordinated family-wide activity, the existing understanding of CHD function in the heart has mostly come from separate, single-factor research studies. Because of this, the literature mostly describes the functions of individual CHD proteins according to their stage or context, and little is known about possible functional integration, redundancy, or division of labor among members of the CHD family.

Individual CHD protein variables have been demonstrated to affect cardiogenic competence by modifying chromatin accessibility at cardiac gene loci during early cardiac lineage specification. While NuRD-associated CHDs impose transcriptional constraint at non-cardiac or alternate lineage genes, enhancer-associated CHDs encourage the activation of cardiac transcriptional regulation [7, 12, 31, 32, 41, 44]. Although these findings highlight the significance of chromatin remodeling in lineage commitment, it remains unclear whether multiple CHD proteins act in a coordinated manner at this stage, given that current insights are largely inferred from studies focusing on single-gene perturbations or isolated epigenomic disruptions. Defects in heart tube remodeling, chamber specification, septation, or outflow-tract development result from disruption of several CHD genes’ function as development progresses into cardiac morphogenesis and chamber creation [3, 6, 16, 20]. These findings demonstrate that precisely timed activation and repression of gene expression programs are essential for proper spatial patterning in the heart. However, if CHD remodelers operate in parallel, sequentially, or in compensatory ways throughout morphogenesis has not been thoroughly investigated. Individual CHD proteins have been connected to the regulation of cell-cycle control, sarcomeric gene expression, metabolic reprogramming, and electrophysiological stability at later stages that include proliferation, differentiation, and maturation. Extensive chromatin reconfiguration is involved in these processes, and CHD-dependent remodeling seems to facilitate functional maturation and stabilize cardiomyocyte identity [12, 13, 22, 42, 56]. However, it is difficult to deduce common or convergent pathways throughout the CHD family due to the variety of experimental settings and single-factor focus of previous research.

Current data points to a scenario in which different CHD chromatin remodelers have stage-specific effects on cardiac development, but also highlights a significant knowledge gap regarding their possible coordination.

### Stage-specific functions and family-level coordination: a working model

Given that most existing studies examine individual CHD-family chromatin remodelers in isolation, coordinated regulation by this protein family in cardiac development should currently be considered a testable hypothesis rather than an experimentally validated mechanism. Nevertheless, accumulating evidence supports several biologically plausible models of functional interaction among CHD remodelers.

A parallel model proposes that distinct CHD remodelers operate in spatially or lineage-restricted contexts during overlapping developmental windows. For instance, CHD7 is involved in neural-crest and second-heart-field enhancer regulation, CHD4 (as part of the NuRD complex) is involved in maintaining myocardial lineage fidelity, and CHD8 is involved in ventricular chamber maturation. A sequential model posits that one remodeler establishes transcriptional competence at an early developmental stage, which is subsequently stabilized or refined by another remodeler acting at a later timepoint. A compensatory model suggests partially redundant functions, particularly between CHD3 and CHD4, that become phenotypically apparent only when both CHD3 and CHD4 are perturbed simultaneously.

All three models are empirically tractable. Experimental strategies including combinatorial genetic modification, inducible and temporally restricted knockout or knockdown approaches, time-resolved single-cell RNA sequencing and ATAC sequencing, high-resolution enhancer mapping, and chromatin occupancy profiling (e.g., chromatin immunoprecipitation sequencing or CUT and RUN) can collectively determine whether CHD-family members converge on shared cis-regulatory elements, function in mutually exclusive cellular lineages, or buffer one another’s activity across key developmental transitions. Such integrative approaches are especially critical at morphogenetic boundaries, where precise spatiotemporal coordination of lineage specification, enhancer activation, and chamber-specific gene silencing is essential.

A stagewise synthesis further underscores the biological coherence of this conceptual framework: acquisition of cardiogenic competence underpins early heart-field specification; accurate lineage allocation and repression of alternative transcriptional regulation govern mid-gestational morphogenesis; and stabilization of contractile, metabolic, and electrophysiological identities characterizes late fetal and postnatal maturation. While CHD remodelers are mechanistically plausible contributors at each of these stages, direct experimental evidence for coordinated, family-level regulation during cardiac development is lacking.

## Experimental models and technologies to study CHD function in the heart

Dissecting stage-specific and cell-type-dependent functions of CHD chromatin remodelers in heart development requires experimental systems that capture both developmental dynamics and chromatin-level regulation. A combination of animal models, human stem-cell-based systems, and emerging multi-omics technologies has substantially advanced our understanding of CHD-dependent mechanisms.

### Animal models

Mouse models have provided critical insights into the in vivo roles of CHD proteins during cardiogenesis. Conventional knockout studies revealed essential functions of CHD7 and CHD8 in embryonic development, although early lethality in some models limits mechanistic resolution [38, 57, 58]. Conditional and tissue-specific knockout strategies, using cardiac progenitor- or cardiomyocyte-specific Cre drivers (Cre: site-specific recombinase used for conditional gene deletion), have enabled temporal dissection of CHD function during lineage specification, morphogenesis, and postnatal maturation [12, 38, 39, 45]. These models have demonstrated that CHD proteins often exert distinct functions at different developmental stages, emphasizing the importance of temporal control in experimental design.

### Human pluripotent stem-cell-based models

Human induced pluripotent stem-cell (hiPSC) and human embryonic stem-cell-derived cardiomyocytes provide a complementary platform for studying CHD function in a human genetic context [59]. More recently, three-dimensional cardiac organoids have emerged as powerful systems that capture multicellular interactions and early morphogenetic events, enabling the study of CHD-dependent regulation of progenitor dynamics, chamber-like patterning, and functional maturation [60–62].

### Multi-omics and genome-editing technologies

Single-cell RNA sequencing and single-cell assay for transposase-accessible chromatin sequencing (single-cell ATAC-seq) have revealed cell-type-specific transcriptional and chromatin accessibility changes associated with CHD modulation [4, 63, 64]. Integration of these datasets with chromatin immunoprecipitation-based assays has allowed mapping of CHD occupancy at enhancers and promoters across developmental stages. Genome-editing approaches, including clustered regularly interspaced short palindromic repeats (CRISPR)/CRISPR-associated protein (Cas)-mediated knockout and precise variant modeling, facilitate causal interrogation of CHD-dependent regulatory elements [12, 38, 39, 42, 45]. Together, these technologies provide a framework for linking chromatin remodeling to transcriptional outcomes and developmental phenotypes.

## Therapeutic implications and future directions

Therapeutically, CHD remodelers are attractive, because they sit upstream of enhancer accessibility, transcription-factor occupancy, and long-range gene-regulatory programs. Delineation of CHD-regulated pathways may identify downstream effectors amenable to intervention, including pathways relevant to cardiomyocyte proliferation, stress responses, contractile organization, or metabolic maturation. Patient-derived hiPSC models and engineered organoids also provide a platform for genotype-specific pharmacological testing and for early evaluation of rescue strategies.

At the same time, therapeutic targeting of ATP-dependent chromatin remodelers faces major limitations. These proteins are haploinsufficient, broadly expressed, and deeply integrated into multi-lineage developmental programs. Pharmacological perturbation, therefore, carries a substantial risk of pleiotropic or off-target chromatin effects. The developmental timing of intervention also matters: a strategy that is theoretically beneficial for postnatal maturation may be harmful during embryogenesis. For these reasons, future translational work will likely depend more on selective pathway modulation or locus-restricted epigenetic editing than on blunt global inhibition or activation of remodeler activity.

## Overview of CHD chromatin remodelers in other developmental processes

Beyond cardiac development, CHD chromatin remodelers play fundamental roles across a wide range of developmental and disease contexts, reflecting their conserved functions in regulating chromatin accessibility, transcriptional robustness, and genome stability. Several CHD-family members have been extensively characterized in non-cardiac systems, where pathogenic variants give rise to distinct but overlapping developmental syndromes and disease phenotypes. CHD1 and CHD2 primarily function as transcription-associated chromatin remodelers that maintain open chromatin states at active promoters and enhancers [23–26, 50, 51, 56, 65]. CHD1 has been most intensively studied in cancer biology, where it modulates transcriptional activity, hormone-receptor signaling, and therapeutic responses [56, 65], while CHD2 plays a prominent role in neurodevelopment, with mutations causing epileptic encephalopathy and synaptic gene dysregulation [51, 66, 67]. These findings highlight their importance in highly transcriptionally active or developmentally plastic cell types. CHD3 and CHD4, as ATPase cores of the NuRD complex, integrate nucleosome remodeling with histone deacetylation to enforce lineage restriction and temporal gene silencing [31]. Disruption of NuRD components leads to severe neurodevelopmental disorders characterized by intellectual disability, craniofacial anomalies, and growth abnormalities, underscoring the essential role of CHD3/4-mediated repression in embryonic patterning and organogenesis [68]. CHD5, CHD6, CHD7, and CHD8 exhibit broader, often tissue-specific functions. CHD5 acts as a tumor suppressor and is required for neural development and spermatogenesis [69, 70], whereas CHD6 participates in enhancer activation, DNA-damage responses, and cellular stress pathways, with pathogenic variants linked to premature senescence syndromes [52, 53, 71, 72]. CHD7 and CHD8 are haploinsufficient regulators with multisystem roles, most notably in neurodevelopmental disorders, such as CHARGE syndrome and autistic spectrum disorder, respectively, reflecting their enhancer-centric control of long-range gene-regulatory networks [12, 38, 39, 47, 48, 55, 59, 73–76]. In contrast, CHD9 remains poorly characterized, with limited evidence for defined developmental roles. Collectively, studies outside the cardiac system establish the CHD family as a core epigenetic framework governing tissue-specific gene regulation, developmental timing, and disease susceptibility.

## Conclusions

Current evidence supports a hierarchical but incomplete view of CHD-family function in the heart. CHD7 has the clearest causal linkage to cardiac morphogenesis; CHD4/NuRD is strongly implicated in myocardial lineage fidelity and chamber specification; CHD8 is an emerging regulator of ventricular growth and postnatal structural–metabolic maturation; and other family members remain largely unexplored in dedicated cardiac systems.

Future progress will depend on integrating cardiac developmental biology with chromatin mechanisms at higher resolution. In practical terms, this means mapping remodeler–transcription factor–enhancer interactions in defined lineages, comparing variant classes across model systems, and testing family-level coordination through combinatorial modification rather than single-gene inference alone.

## Data Availability

No datasets were generated or analyzed during the current study.
